# Unprotected left main revascularization: Percutaneous coronary intervention versus coronary artery bypass. An updated systematic review and meta-analysis of randomised controlled trials

**DOI:** 10.1371/journal.pone.0179060

**Published:** 2017-06-28

**Authors:** Luca Testa, Azeem Latib, Mario Bollati, Rocco Antonio Montone, Antonio Colombo, Filippo Crea, Francesco Bedogni

**Affiliations:** 1 Dept. of Cardiology, IRCCS Pol S. Donato, Milan, Italy; 2 Interventional Cardiology Unit, San Raffaele Scientific Institute; EMO GVM Centro Cuore Columbus Milan, Italy; 3 Dept of Cardiology, Catholic Univ. of the Sacred Heart, Rome, Italy; Medstar Washington Hospital Center, UNITED STATES

## Abstract

**Background:**

The optimal treatment of unprotected left main (UPLM) with either PCI or CABG remains uncertain.

**Aim:**

The purpose of this study was to determine the comparative safety and efficacy of PCI versus CABG in patients with UPLM disease.

**Methods:**

Search of BioMedCentral, CENTRAL, mRCT, PubMed, major cardiological congresses proceedings and references cross-check (updated November 2016). Outcomes were the rate of MACE [all cause death, MI, stroke], the rates of the individual components of MACE and the rate of target vessel revascularisation (TVR).

**Results:**

We identified 6 Randomised Controlled Trials totalling 4717 patients allocated to PCI or CABG. At 1 year follow up, PCI and CABG were substantially equivalent with respect to the rates of MACE [PCI 8.5% vs CABG 8.9%, OR 1.02,(0.76–1.36), p = 0.9], death [PCI 5.4% vs CABG 6.6%, OR 0.81,(0.63–1.03),p = 0.08] and MI [PCI 3.4% vs CABG 4.3%, OR 0.80(0.59–1.07), p = 0.14]. Notably, PCI was associated with a significantly lower rate of stroke [PCI 0.6% vs CABG 1.8%, OR 0.36,(0.17–0.79), p = 0.01] and with a significantly higher rate of TVR [PCI 8.7% vs CABG 4.5%, OR 2.00(1.46–2.75), p<0.01]. At a median follow up of 5years, the rates of MACE were similar between the two strategies: PCI 14.6% vs CABG 13.8%, OR 1.02(0.76–1.38), p = 0.89. Likewise, the rates of death [PCI 8% and CABG 7.7%, OR 1(0.77–1.31), P = 0.9], MI [PCI 6.1% vs CABG 5%, OR 1.41(0.85–2.34), P = 0.19, I^2^ 59%], and stroke [PCI 2% vs CABG 2.2%, OR 0.85(0.42–1.81), p = 0.65,] were similar while PCI was associated with a significantly higher rate of TVR [14.5% vs CABG 8.9%, OR 1.73(1.41–2.13), p<0.01].

**Conclusion:**

In patients with UPLM disease, PCI and CABG are associated with similar rates of MACE and mortality at 1 year as well as after 5 years. Differences can be detected for individual end points at both short and long term FU.

## Introduction

European and U.S. guidelines currently recommend that the majority of patients with unprotected left main coronary artery disease (UPLM) undergo coronary-artery bypass grafting (CABG). [1,2]

PCI should be considered in patients with UPLM and coronary disease favorable to PCI (ie, in the absence of complex and diffuse lesions) [1,2] The guidelines are substantially based on the 705 patient subgroup with UPLM in the SYNTAX trial, [3] and on the findings of some randomized trials, LE MANS (100 patients), [4] PRECOMBAT (600 patients), [5] and Boudriot and colleague*s* (201 patients) [6]. Of note, after the publication of the Guidelines, two large randomised controlled trials [7,8] and long term data of the previous studies [9–11] have been published. Thus, we sought to perform an updated meta-analysis of available evidence.

## Methods

### Data sources and searches

BioMedCentral, CENTRAL, mRCT, and PubMed were searched (updated to May 2016), according to an established method [12]. Pertinent studies were also searched in major recent international cardiology meetings. References of original and review articles were cross-checked. The following key words were specifically used for the PuBMed search: “left main” AND “percutaneous coronary intervention” AND “coronary artery bypass graft”.

### Study selection

Characteristics of included studies were: 1) randomised allocation to PCI or CABG to treat UPLM disease, 2) safety and efficacy data reported, 3) complete reporting of procedural and follow up raw data. We evaluated the quality of each included trial by checking for confounders, measurement of exposure, completeness of follow-up, and blinding. No formal scoring system was used. Reviewers were not blinded to journal, authors, or institution of publication.

### Data extraction and endpoints

Two independent reviewers (TL, AL) performed data abstraction. Divergences were resolved by consensus and/or another reviewer. Primary end point was the MACE rate defined as combination of overall mortality, stroke, myocardial infarction. Data concerning the Target vessel revascularisation [TVR] rate were also appraised. Whereas not clearly stated, estimates from Kaplan Meier curves were visually derived to obtain crude numbers of end-points.

Additional analyses were carried out for single end points. One year and longest available follow up data have been appraised and analysed.

### Validity assessment

Two unblinded reviewers (TL, AL) appraised the internal validity of included studies, none involved in any of the included studies, with divergences resolved by consensus, according to the methods of The Cochrane Collaboration [13]. Specifically, we adjudicated explicitly the risk for selection, performance, attrition, and adjudication biases, and expressed as low risk of bias (A), moderate risk of bias (B), high risk of bias (C), or incomplete reporting leading to inability to ascertain the underlying risk of bias (D).[13]

### Data synthesis and analysis

Statistical analysis was performed using Review Manager version 5.3 (Copenhagen: The Nordic Cochrane Centre, The Cochrane Collaboration, 2014). [14].

Binary outcomes from individual studies were combined with Der Simonian and Laird random-effect model, according to an “intention to treat” analysis to obtain odds ratios (OR) with 95% confidence intervals (95%CI) and confirmed by a fixed-effects model to avoid overweighting of small studies. We also carried out the “z” test with z = estimated effect size / standard error of the estimated effect size, and the odds ratio considered on the log scale. As log(OR) has a unimodal distribution, the reported z values were analysed to obtain a two-tailed “p”, and hypothesis testing results were considered statistically significant at the 0.05 level [13].

We computed Cochrane Q heterogeneity test (H) by summing the squared deviations of each study’s estimate from the overall meta-analytic estimate, weighting each study’s contribution in the same manner. Heterogeneity was considered significant at “p for H” <0.10 [13].

In order to incorporate between-study variation in estimating summaries of effect size, the choice between random effect and fixed effect models usually makes little difference in the results if there is little heterogeneity and inconsistency. In presence of significant heterogeneity, it may be more appropriate to analyze results using both methods as the fixed effect model indicates that there is an effect in at least one of the studies, and the overall result is an average measure of treatment effect across all the studies, while, on the other hand, the random effects approach relies on assumptions that the studies are a random sample from a hypothetical population of studies and that the heterogeneity between studies can be represented by a single variance. The random effects model tends to give a more conservative estimate (i.e. with wider confidence intervals) thus, we calculated the cumulative OR with both models and no significant differences were found, however, we presented the results according to the Random Effect. We also performed a meta-regression analysis to assess the potential effect of age, male gender, diabetes, acute coronary syndrome at presentation, mean left ventricular ejection fraction, distal LMCA involvement/bifurcation/trifurcation lesion, mean SYNTAX score, mean logistic EuroSCORE, complete revascularization, or use of first-generation DES. A subgroup analysis according to SYNTAX scores has been performed.

The likelihood of publication bias was assessed graphically by generating a funnel plot for the combined endpoint of MACE and mathematically by means of Egger’s test (p for significant asymmetry <0.1) [15].

This study is inspired by good practice guidelines [16], including those from the Cochrane Collaboration, and the Quality of Reporting of Meta-analyses (QUOROM) statement [16].

## Results

### Search results

**Fig 1:**
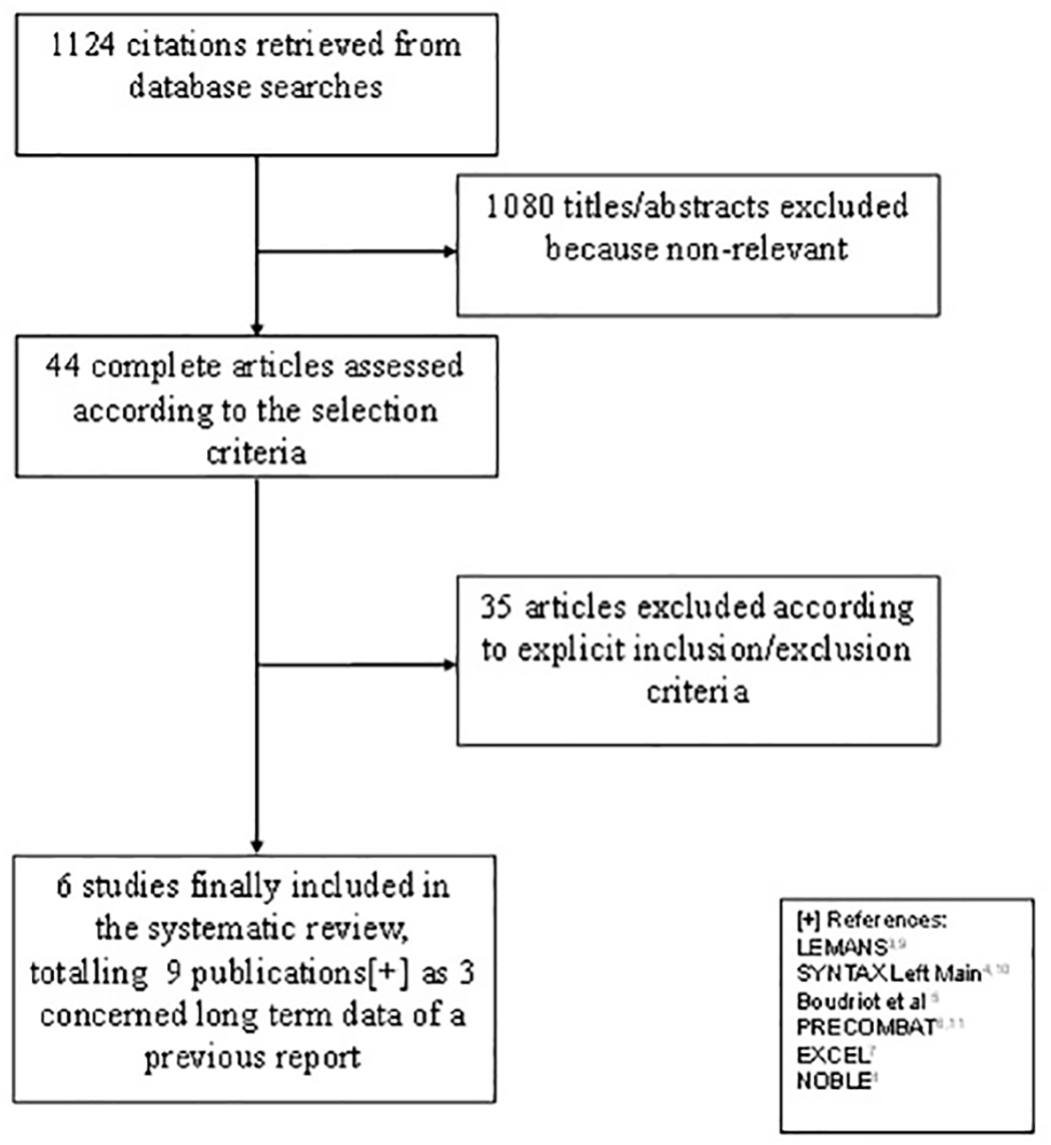
Flow diagram of study selection.

As shown in Fig 1, 1124 potentially eligible studies were identified, 6 of which met the pre-specified inclusion criteria (Tables 1 and 2). Three studies reported short and long term data separately [3,4,6,9–11].Five studies were RCTs comparing PCI and CABG in UPLM disease [3,5–8]. One study was a pre-specified subanalysis of the SYNTAX (Synergy Between PCI With Taxus and Cardiac Surgery) trial [4]. Of 4686 randomized patients, 2347 were assigned to PCI and 2339 were assigned to CABG. First-generation drug eluting stents (DES) were implanted in 775 patients (33%), while second generation DES in 1546 patients (66%). An arterial conduct to the left anterior descending artery was used in 2264 patients treated with CABG (96%). Distal LM involvement was observed in 74.6% of cases. In the first 4 studies (LEMANS [3,9], SYNTAX Left Main [4,10], Boudriot et al [5]. and PRECOMBAT [6,11]), the mean SYNTAX score ranged from 24 to 30. In the EXCEL[7] and NOBLE[8] trials, which have been designed after the publication of the SYNTAX trial, the mean SYNTAX score was 20.5 (SD 6.2), and 22.4 (SD 7.7) respectively.

**Table 1:** Features of included studies. CABG: Coronary artery bypass graft; CVA; cerebrovascular accident; DES: drug eluting stent; LAD: left anterior descending; MI: myocardial infarction; PCI: percutaneous coronary intervention; RCT: randomised controlled trial.

Study	Design	PCI (N)	DES, %	CABG (N)	Arterial graft to LAD, %	Primary end point
LEMANS^3,9^	RCT	52	35	53	81	Cardiac death, MI, CVA, repeat revascularization, and/or acute/subacute ST
SYNTAX Left Main^4,10^	Pre-specified subanalysis from a RCT	357	100	348	97	All-cause death, CVA, MI, and repeat revascularization
Boudriot et al ^5^	RCT	100	100	101	99	All-cause death, MI, and repeat revascularization
PRECOMBAT^6,11^	RCT	300	100	300	94	All-cause death, CVA, MI, and repeat revascularization
EXCEL^7^	RCT	948	100	957	99	Death, stroke, or myocardial infarction
NOBLE^8^	RCT	598	100	603	93	Death from any cause, non-procedural myocardial infarction, repeat revascularisation, or stroke

**Table 2:** Patients and procedural features of included studies.

Study	Age	Diabetes	Distal LM	No of Diseased vessel 0/1/2/3%	Syntax Score	Complete Revascularization (overall,PCI,CABG)
LEMANS^3,9^	61	18	58	0/9/23/68	23	84/79/89
SYNTAX Left Main^4,10^	65	25	61	13/20/31/36	30	68/65/73
Boudriot et al ^5^	68	36	71	29/31/27/14	24	98/98/97
PRECOMBAT^6,11^	62	32	65	10/17/32/41	25	69/68/70
EXCEL^7^	66	29	80	163/292/325/162	20	NA
NOBLE^8^	66	15	81	NA	22	92 (PCI only)

### Clinical outcomes meta-analysis at 1 year follow up

**Fig 2:**
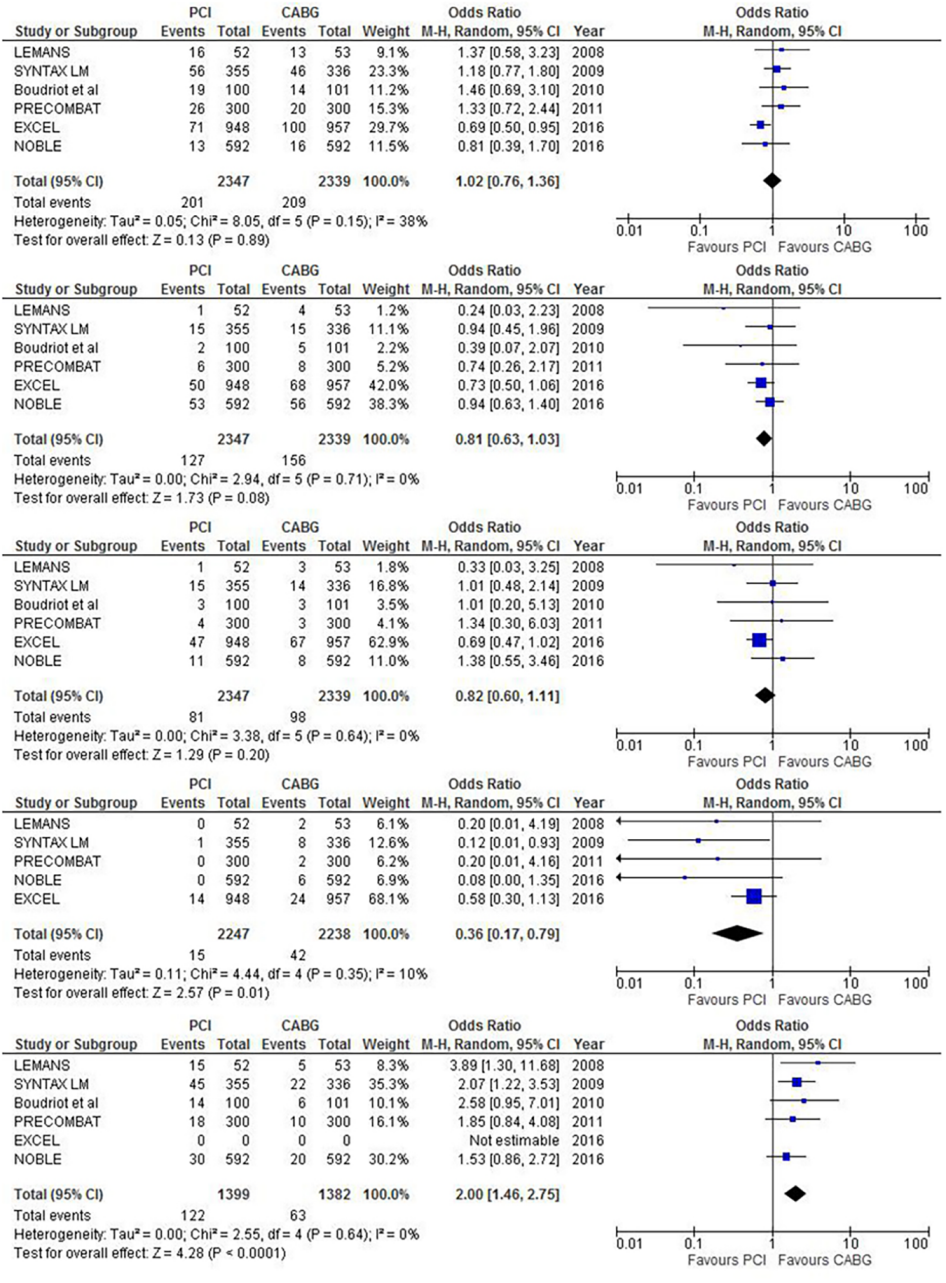
Major adverse cardiovascular events at 1 year (from top to bottom, MACE, Death, MI, Stroke, TVR).

PCI and CABG (Fig 2 and Table 3) were substantially equivalent with respect to MACE [PCI 8.5% vs CABG 8.9%, OR 1.02, (0.76–1.36), p = 0.9, I^2^ = 38], overall death [PCI 5.4% vs CABG 6.6%, OR 0.81, (0.63–1.03), p = 0.08, I^2^ 0%], and MI [PCI 3.4% vs CABG 4.3%, OR 0.80(0.59–1.07), p = 0.14]. Notably, PCI was associated with a significantly lower risk of stroke [PCI 0.6% vs CABG 1.8%, OR 0.36,(0.17–0.79), P = 0.01, I^2^ = 10%, NNT: 100] and a significantly higher risk of TVR [PCI 8.7% vs CABG 4.5%, OR 2.00 (1.46–2.75), P<0.01, I^2^ 0%, NNH: 20].

**Table 3:** One year rate of clinical events (P refers to pooled OR).

Study	PCI (%)	CABG (%)	Absolute Difference	P value
MACE	8.5	8.9	0.4	0.9
Death	5.5	6.6	1.2	0.07
MI	3.4	2.6	0.9	0.14
Stroke	0.6	1.8	1.2	0.01
TVR	8.7	4.5	4.2	<0.01

### Clinical outcomes meta-analysis beyond 1 year follow up

**Fig 3:**
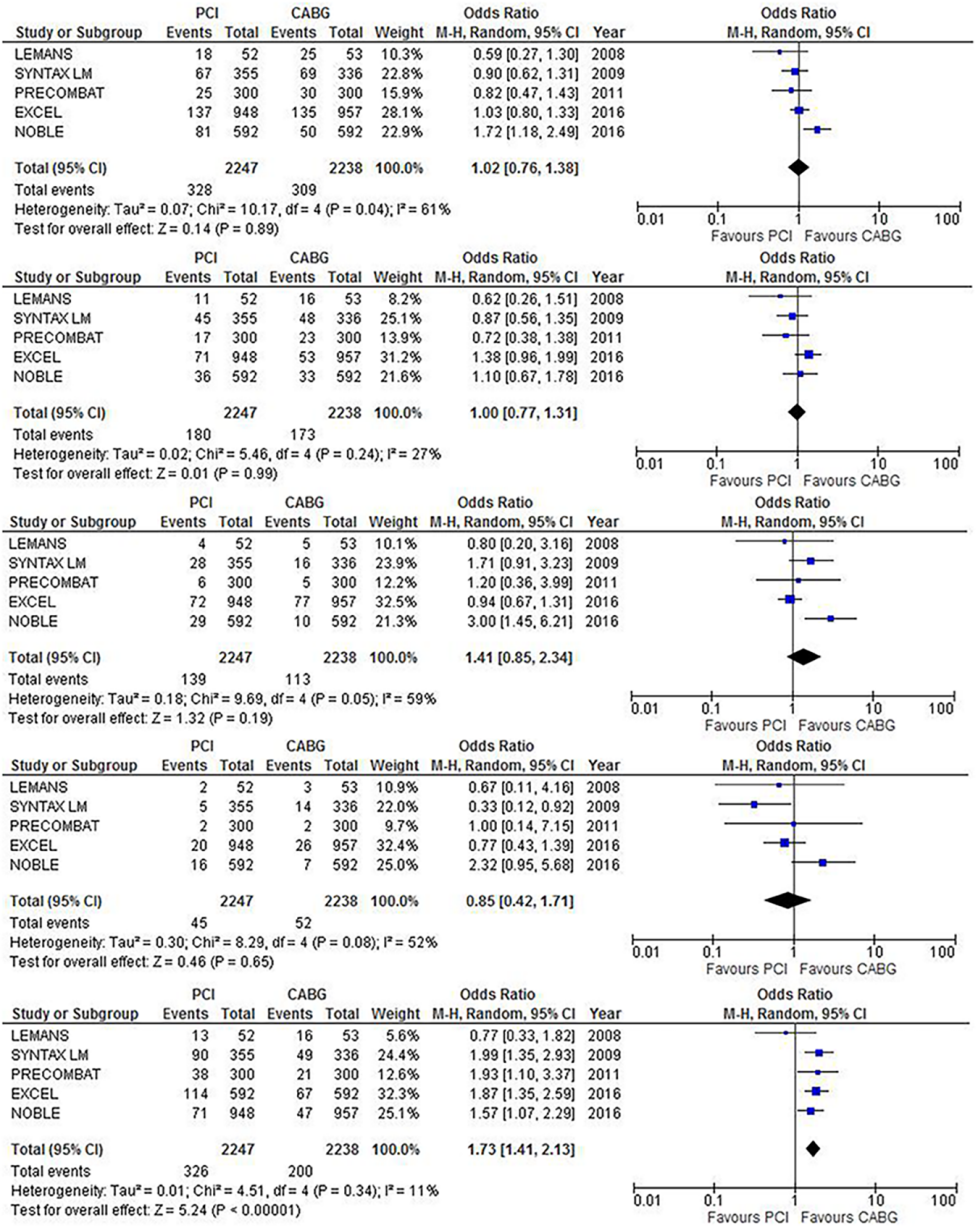
Major adverse cardiovascular events at a median FU of 5 years, mean 5.6 years (from top to bottom, MACE, Death, MI, Stroke, TVR).

At a median follow up of 5 years, (Fig 3 and Table 4), the rate of MACE was similar between the two strategies: PCI 14.6% vs CABG 13.8%, OR 1.02 (0.76–1.38), p = 0.89, I^2^ 61%. Likewise, the rate of overall death [PCI 8% and CABG 7.7%, OR 1(0.77–1.31), P = 0.9, I^2^ 27%], MI [PCI 6.1% vs CABG 5%, OR 1.41 (0.85–2.34), P = 0.19, I^2^ 59%], and stroke [PCI 2% vs CABG 2.2%, OR 0.85(0.42–1.81), p = 0.65, I^2^ 52%] did not significantly differ between the two strategies. PCI was associated with a significantly higher rate of TVR 14.5% vs CABG 8.9%, OR 1.73 (1.41–2.13), p<0.01, I^2^ 11%, NNH 16.

**Table 4:** Clinical events rate at a median FU of 5 years (P refers to pooled OR).

Study	PCI (N:)	CABG (N:)	Absolute Difference	P value
MACE	14.6	13.8	0.8	0.8
Death	8	7.7	0.3	0.9
MI	6.1	5	1.1	0.1
Stroke	2	2.2	0.2	0.65
TVR	14.5	8.9	5.6	<0.01

#### Subgroup analysis according to SYNTAX scores

Three trials (EXCEL, SYNTAX, and bypass surgery versus angioplasty using sirolimus-eluting stent in patients with left main coronary artery disease [PRECOMBAT]) reported MACE stratified by number of coronary vessels involved. In pooled analyses of these 3 trials, the MACE rates were similar with PCI and CABG for isolated LMCA disease, LMCA plus 1-vessel disease, and LMCA plus 2- vessel disease. A trend of higher MACE was observed with PCI in patients with LMCA plus 3-vessel disease. No significant difference for MACE was observed for distal LMCA lesions including bifurcation/trifurcation involvement. The rate of MACE was similar between DES and CABG group in the lower 2 SYNTAX score tertiles (scores between 0 and 32). In the subgroup of patients with high SYNTAX scores (≥33), the risk of MACE was not significantly different between the 2 treatment arms; however, repeat revascularization was significantly greater in the PCI group.

### Meta regressions

Meta-regression analysis did not disclose statistically significant interactions for any of the outcomes between PCI versus CABG and the log-OR of the number of enrolled diabetic patient, distal LMCA involvement, mean SYNTAX score, age, clinical presentation, use of II generation stents, use of arterial mammary, or complete revascularization (all p nonsignificant).

### Validity and systematic bias assessment

**Fig 4:**
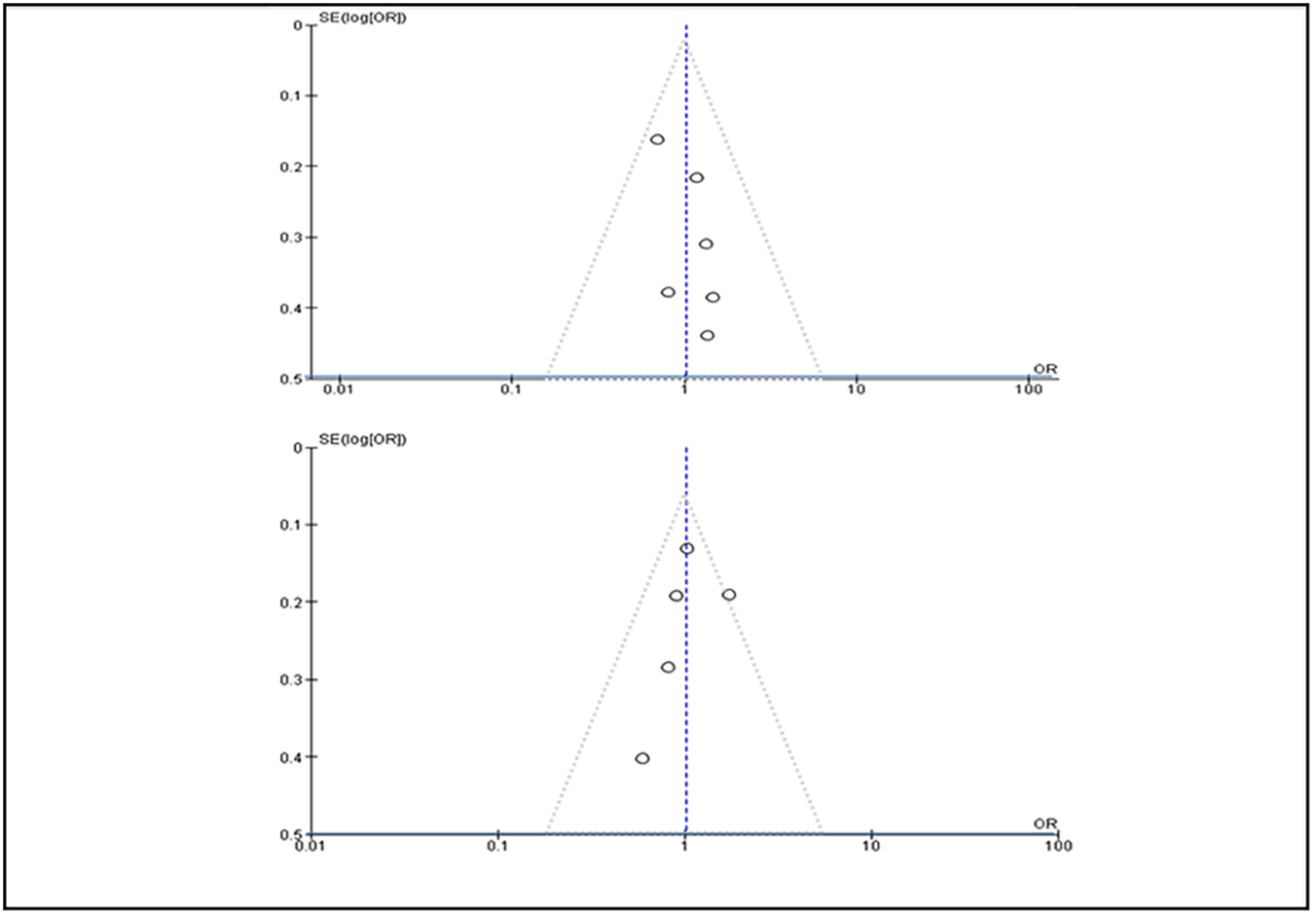
Funnel plot of MACE at 1 year and long term FU.

The overall internal validity of all included studies was good, although the risk of Performance and Detection biases was moderate because of unclear blinding of Clinical Event Committees (Table 5). Moreover, the risk of systematic bias, as assessed by visual estimation of funnel plots for 1 year MACE and long term MACE (Fig 4) as well as by means of Egger’s test was ruled out (p = 0.14). Sensitivity analysis showed that the exclusion of one study at a time did not significantly alter the cumulative ORs, thus ruling out the possible impact of differences in terms of end point definitions across included studies.

**Table 5:** Design features and appraisal of the internal validity of included studies. Risk of bias is expressed as A (low risk), B (moderate risk), C (high risk), and D (incomplete reporting leading to inability to ascertain the underlying risk of bias).

Study	Prospective design	Multicenter enrolment	Selection bias	Performance bias	Attrition bias	Detection bias	Multivariable adjustment for potential confounders
LEMANS^3,9^	YES	YES	A	B	B	B	Probably adequate
SYNTAX Left Main^4,10^	YES	YES	A	B	B	B	Probably adequate
Boudriot et al ^5^	YES	YES	A	B	B	B	Probably adequate
PRECOMBAT^6,11^	YES	YES	A	B	B	B	Probably adequate
EXCEL^7^	YES	YES	A	B	B	B	Probably adequate
NOBLE^8^	YES	YES	A	B	B	B	Probably adequate

## Discussion

The present meta-analysis summarises current evidences from all the randomised controlled trials concerning the treatment of UPLM with either PCI or CABG at both short and long term follow up.

Main findings can be featured as follows:

- At 1 year follow-up PCI and CABG achieve substantial equivalence with respect to the cumulative end point of MACE (overall death, MI and stroke), while CABG is associated with a higher risk of stroke and PCI is associated with a higher risk of TVR
- At long term follow up, PCI and CABG are equivalent with respect to MACE, mortality, MI and risk of stroke, while PCI is associated with a higher risk of TVR.

Current U.S. multidisciplinary guidelines give a general indication to CABG for all those conditions in which revascularisation of a complex coronary artery disease, especially when involving the proximal left anterior descending (LAD) artery, can improve the survival [1]. European guidelines give a class IB indication to CABG for the treatment of UPLM, regardless the extent of coronary artery disease; class IB to PCI only in the case of SYNTAX score ≤ 22, class IIa for Syntax score 23–32, and class IIIB for Syntax score >32 as well as for LM disease plus 2 or 3 vessel disease [2].

A previous meta-analysis [17], already suggested that 1 year MACE rate was similar for PCI and CABG, with an advantage of PCI for risk of stroke and an advantage of CABG for risk of TVR. However, authors could not pool long term data and EXCEL [7] and NOBLE[8] trials were still enrolling.

A meta-analysis including the EXCEL and NOBLE trial has been recently published [18], however, authors did not include the LEMANS trial neither at short nor at long term [3,9] and did not check the results using both random and fixed effect models. Of note, in the calculation of the MACE, authors did not include the NOBLE trial because the primary end point was differently defined. This had cut > 1000 patients from the analysis. Of note, the single end points are actually available in the manuscript [8], in order to calculate the MACE as defined by the authors of the meta-analysis. Finally, they did not distinguish end points at short and long term follow up, which is of outmost relevance in order to provide robust evidence of the true effect of the two strategies.

All the studies included in the present meta-analysis were designed for non-inferiority and, taken singularly, may have been underpowered to detect true differences for clinical events, thus, pooling 4717 patients may overcome these limitations. Moreover, the management of patients in these trials reflects the current practice as almost all patients were treated with drug eluting stents, perhaps second generation in the EXCEL and NOBLE trials, while the vast majority of CABG patients received an arterial conduct to the LAD.

Of note, both EXCEL and NOBLE have been designed after the SYNTAX trial and aimed to enrol patients at low to moderate risk according to SYNTAX score. However, the EXCEL trial enrolled 239 patients with a Syntax score ≥33 after central lab angiographic evaluation and results were consistent with those of a similar subgroup (284 patients) in the SYNTAX trial [18], i.e. the risk of MACE was similar between PCI and CABG. The risk of cardiac mortality was significantly higher in the PCI arm in the high risk subgroup of SYNTAX but not in the subgroup of EXCEL, that used everolimus eluting stents instead of paclitaxel eluting stents [19].

Although associated with a significantly higher risk of stroke at short term, CABG was associated, in this meta-analysis, with a lower risk of TVR as compared to PCI, at both short and long term. Yet, in some patients and doctors view, considering the longer stay in hospital, the risk of reoperation for bleeding and infection, and a longer recovery time, the surgical approach might not be worth the lower risk of TVR as no difference in mortality and MACEs can be detected. Any generalization about the management of patients with LM disease can be misleading if not translated to the single anatomical and clinical condition; accordingly all patients with complex multivessel coronary artery disease involving the LM should be reviewed and discussed by a clinician, a cardiac surgeon and an interventional cardiologist to reach consensus on optimum treatment, considering the conundrum of clinical setting, anatomy, physiology, technical challenges and operators/center experience as well as patient preferences. Of note, the operators experience and cath lab volume is one of the major obstacle for PCI to be considered a mainstream therapy for the UPLM, in particular if it is associated with severe CAD, although recent evidences suggest a very reassuring long term safety and efficacy profile[20] In other words, PCI can be truly challenging in some patients and, thus, results can be strongly conditioned by the operator skills and experience, while the results of CABG are actually more predictable even in low-medium volume surgical centers.

### Limitations of the present study

A limitation inherent to all meta-analyses is the potential heterogeneity among studies, in terms of protocols, patients, end point definitions, sample sizes, and the unavailability of patient-level data.

Sensitivity analysis showed that the main results of the studies were actually consistent, although some differences in terms of end point definitions must be acknowledged, i.e. the composite primary MACE (or MACCE) endpoints were similar in the NOBLE trial, the SYNTAX trial and the EXCEL trial except that NOBLE did not include peri-procedural myocardial infarction and in the EXCEL trial authors have chosen a single definition for both approaches in order to reduce the risk of ascertainment bias (a rise in the level of the MB fraction of creatine kinase to more than 10 times the upper reference limit of the assay or more than 5 times if additional angiographic, electrocardiographic, or imaging evidence of infarction was present). Studies have been performed with similar but not identical techniques and with both first and second generation DES, so the results could be affected by the DES type.

## Conclusion

The present manuscript summarises available evidence concerning the UPLM disease treatment while further large scale randomised controlled trials appear to be unlikely. The prognosis of patients with UPLM can be successfully improved with either PCI or CABG as no significant differences could be detected with respect to hard MACE. All patients with complex coronary artery disease involving UPLM should be carefully evaluated by the Heart Team, which is the appropriate setting to discuss these challenging patients.

## Supporting information

S1 Table PRISMA checklist. (DOC)

## Abbreviations

CABG: coronary artery bypass graft
PCI: percutaneous coronary intervention
UPLM: unprotected left main
